# The Tip Region on VP2 Protein of Bluetongue Virus Contains Potential IL-4-Inducing Amino Acid Peptide Segments

**DOI:** 10.3390/pathogens10010003

**Published:** 2020-12-22

**Authors:** Jia-Ling Yang, Chia-Yi Chang, Chih-Shuan Sheng, Chia-Chi Wang, Fun-In Wang

**Affiliations:** 1 School of Veterinary Medicine, National Taiwan University, No. 1, Section 4, Roosevelt Road, Taipei 10617, Taiwan; yukirokira@gmail.com (J.-L.Y.); meowcat01@gmail.com (C.-S.S.); 2 OIE Reference Expert for CSF, Animal Health Research Institute, Council of Agriculture, Executive Yuan, 376 Chung-Cheng Road, Tansui District, New Taipei City 25158, Taiwan; cychang@mail.nvri.gov.tw

**Keywords:** bluetongue virus (BTV), bioinformatics, cytokines, IL-4, monocyte-derived macrophage (MDM), peripheral blood mononuclear cell (PBMC), pathogenesis

## Abstract

Bluetongue is an infectious viral hemorrhagic disease of domestic and wild ruminants that has a considerable economic impact on domestic ruminants. There are currently at least 29 serotypes of bluetongue virus (BTV) in the world. Noteworthily, the pathogenesis among BTV serotypes is different, even in the same animal species. In this study, BTV2/KM/2003 and BTV12/PT/2003 were used to investigate the differential immunological effects on bovine peripheral blood mononuclear cells (PBMCs). The BTV viral load and the expression of cytokine messenger RNA (mRNA) in PBMCs were measured by fluorescence-based real-time reverse-transcription PCR (qRT-PCR). The immunofluorescence assay (IFA) was applied to detect BTV signals in monocyte-derived macrophages (MDMs). The SWISS-MODEL and IL-4pred prediction tools were used to predict the interleukin 4 (IL-4)-inducing peptides in BTV-coat protein VP2. Synthetic peptides of VP2 were used to stimulate PBMCs for IL-4-inducing capability. This study demonstrated that the cytokine profiles of BTV-induced PBMCs were significantly different between BTV2/KM/2003 and BTV12/PT/2003. BTV2 preferentially activated the T helper 2 (Th2) pathway, represented by the early induction of IL-4, and likely fed back to inhibit the innate immunity. In contrast, BTV12 preferentially activated the innate immunity, represented by the induction of tumor necrosis factor -α (TNF-α) and interleukin 1 (IL-1), with only minimal subsequent IL-4. The BTV nonstructural protein 3 antibody (anti-BTV-NS3) fluorescent signals demonstrated that monocytes in PBMCs and MDMs were the preferred targets of BTV replication. Bioinformatics analysis revealed that the capability to induce IL-4 was attributed to the tip region of the VP2 protein, wherein a higher number of predicted peptide segments on BTVs were positively correlated with the allergic reaction reported in cattle. Synthetic peptides of BTV2-VP2 induced significant IL-4 within 12–24 h post-infection (hpi) in PBMCs, whereas those of BTV12 did not, consistent with the bioinformatics prediction. Bovine PBMCs and synthetic peptides together seem to serve as a good model for pursuing the BTV-induced IL-4 activity that precedes the development of an allergic reaction, although further optimization of the protocol is warranted.

## 1. Introduction

Bluetongue (BT) is an important viral disease of domestic and wild ruminants. In the livestock industry, BT causes considerable economic loss, which affects national and international trade. It is caused by the bluetongue virus (BTV) and transmitted by *Culicoides* biting midges. Clinical signs of BT are commonly observed in sheep, white-tailed deer, cattle, and other species. At least 29 distinct serotypes currently exist worldwide [1]. Since 1998, several incursions of different BTV serotypes have occurred in the Mediterranean Basin. The appearance and spread of BTV8 also began in Northwestern Europe in 2006, causing severe disease not only in sheep but also in cattle populations [2]. 

Since the immunogenicity differs among BTV serotypes, the host immune responses they induce are also different [3]. For example, higher interleukin-1 (IL-1) protein expression was detected in BTV1-infected sheep than in those infected with BTV8, and this higher IL-1 expression is likely associated with more severe clinical symptoms and lesions [4]. Another example is that BTV11 and BTV17 induced immunoglobulin-E (IgE) production in the peripheral blood mononuclear cells (PBMCs) of experimentally infected animals [5,6] and presumably contributed to type I hypersensitivity. Similarly, the BTV2/KM/2003 Taiwanese strain causes significant IL-4 expression and IgE secretion [7]. The underlying mechanisms of how different serotypes of BTV induce differential host immune responses remain unclear.

BTV is a non-enveloped, icosahedral, double-stranded RNA (dsRNA) virus, which belongs to the Reoviridae family and *Orbivirus* genus. The BTV genome is composed of 10 segments of dsRNA and encodes seven structural viral proteins (VP1–VP7) and four non-structural proteins (NS1–NS4). BTV virions are comprised of outer (VP2 and VP5) and inner (VP3 and VP7) capsids. Proteomics studies indicate that VP2 has an accurate interaction with VP5 and VP7. These three proteins precisely regulate the entry process of the virus into host cells [8,9]. They are also the major proteins that induce type I hypersensitivity in cattle, especially VP5, which is the strongest inducer of the IgE antibody [5]. In this study, two Taiwan isolates (BTV2/KM/2003 and BTV12/PT/2003), which are epidemic wild-type strains, were used to study the potential roles of VP2, VP5, and VP7 in host immunity. Analysis of the amino acid sequence in each segment of these two Taiwan isolates showed that all viral proteins are very similar, with at least 96.8% nucleotide similarity, except for the VP2 protein (only 25.8% similarity in amino acids) [10]. We hypothesized that VP2 is probably the key factor in the induction of differential immune responses in the host. 

To explore this issue further, two immune protein databases were utilized to predict whether the BTV VP2 protein contains the potential IL-4-inducing peptide segments. The triskelion shape of the VP2 protein contains three tip domains and a central hub domain to form a sialic acid binding pocket, which is used to enter the host cells by receptor-mediated endocytosis [8]. At the same time, the VP2 protein is also cleaved by lysozyme and presented to CD4^+^ T cells by major histocompatibility complex (MHC) class II molecules [8]. The currently used database platform is the Immune Epitope Database and Analysis Resource (IEDB), providing a binding peptide prediction service for cattle MHC class I. However, the IEDB only has an analytical database for human MHC class II binding protein prediction. Since the MHC class II molecular similarity between human and bovine samples is high [11,12], we hypothesized that the amino acid fragments presented by cattle antigen-presenting cells (APC) may resemble those in humans. The prediction of MHC binding amino sequences from IEDB will be used to explore the potential immunogenic peptides of two Taiwan isolates in this study. 

In our recent study, the BTV2/KM/2003 induced significant IL-4 expression in bovine PBMCs [7]. As IL-4 is the key cytokine for the induction of type I hypersensitivity, the potential capability of viral peptides to induce IL-4 is an important event. Although the amino acid sequence similarity of VP2 between BTV2 and BTV12 is low, their three-dimensional (3D) structure is roughly similar. There are four parts of the VP2 protein: the body, hairpin, hub, and tip regions [9]. The tip region is the most different area among serotypes [9]. The VP2 of other strains will be analyzed to find regularity by a bioinformatics approach. The IL-4-inducing peptide analysis database (IL-4pred) [13] will be utilized to predict potential IL-4 inducing peptides in the tip regions (191-407a.a.) of VP2 in this study. 

BTV has been shown to be a strong inducer of type I interferon (IFN) in host immunity [14]. It is also able to modulate the type I IFN production pathway [15,16]. The nonstructural protein NS3 inhibited the type I IFN by interfering with the IFN-I synthesis pathway downstream of retinoic acid-inducible gene I (RIG-I) and upstream of TANK-binding kinase 1/IκB kinase ε (TBK1/IKKε) activation [15]. The nonstructural protein NS4 inhibited the type I IFN by downregulating the activities of a variety of promoters, such as the cytomegalovirus immediate-early promoter and a promoter containing interferon-stimulated response elements (ISRE) [16]. Noteworthily, the NS4 amino acid sequences of Taiwanese strains [10] shared 94.8% (BTV2) and 92.2% (BTV12) nucleotide similarities with the NS4 consensus sequence of the reference [16]. The effects of these Taiwanese strains on type I IFN production will be determined in the study.

The aim of this study was to explore whether the two BTV strains of BTV2/KM/2003 and BTV12/PT/2003 induce different immune responses in bovine PBMCs. The potential IL-4-inducing peptide segments on VP2 of BTV2 (allergy reported) and BTV12 (non-allergy reported) were synthesized to test for their ability to induce IL-4 in bovine PBMCs. This combination of protein immunogenicity prediction and experimental cytokine profiles may provide a new breakthrough in the study of BTV pathogenesis.

## 2. Results

### 2.1. Experimental Design

**Figure 1:**
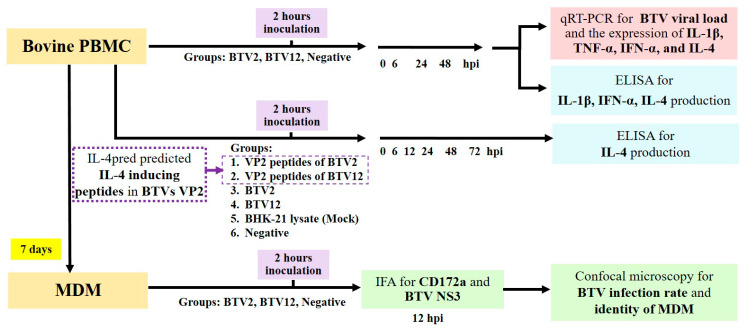
The experimental design. The experimental timeline and the detection parameters and methods are briefly demonstrated. PBMC, peripheral blood mononuclear cell; MDM, monocyte-derived macrophage; BTV, bluetongue virus; TNF, tumor necrosis factor; IFN, interferon; IL, interleukin; hpi, hours post-infection.

To demonstrate the differential immune responses of bovine PBMCs to BTV2 and BTV12, PBMCs were infected and harvested at various hours post-infection (hpi). The expression of messenger RNA (mRNA) of IL-1β, tumor necrosis factor -α (TNFα), IFNα, and IL-4 was quantified by real-time reverse-transcription PCR (qRT-PCR). The protein levels of IL-1β, IFNα, and IL-4 in the culture supernatants were assayed by an enzyme-linked immunosorbent assay (ELISA). Bioinformatics analysis (through the IL-4pred database) revealed that the ability of BTV2 to induce significant IL-4 resides in the tip region of VP2, which has a higher number of potential IL-4-inducing peptide segments. Then synthetic IL-4-inducing peptide segments of BTV2 (and of BTV12 on the corresponding locations for comparison) were used to stimulate PBMCs. The culture supernatants of stimulated and infected PBMCs were quantified for the protein levels of IL-4. To confirm the identity of monocyte-derived macrophages (MDMs) and the infection yields, MDMs at day 7 of cultivation were infected with either BTV2 or BTV12 and harvested at 12 hpi. MDMs were labeled by using the macrophage surface marker antibody (anti-CD172a) and BTV nonstructural protein 3 antibody (anti-NS3) in an immunofluorescence assay (IFA) before being counted for the infection rates (Figure 1).

### 2.2. The Viral Loads and Titers of BTV2/BTV12 in Infected PBMCs

**Figure 2:**
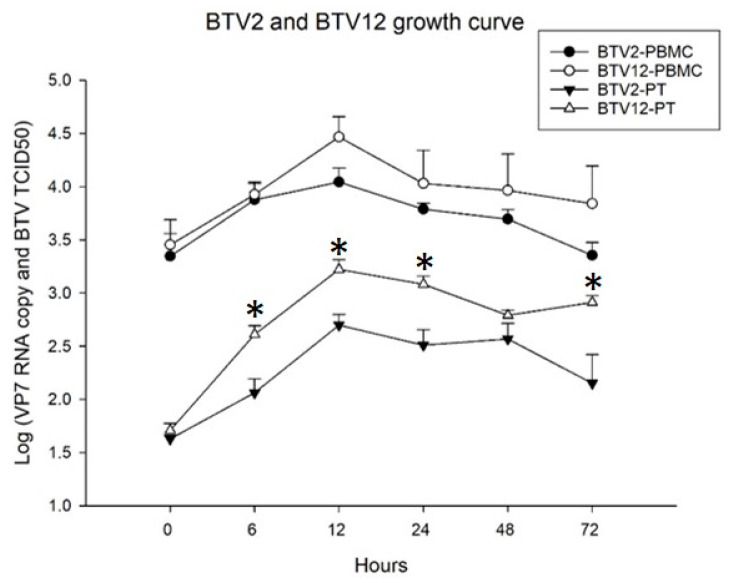
Bluetongue virus (BTV) growth in peripheral blood mononuclear cells (PBMCs). Absolute quantification for the intracellular VP7 RNA expression (upper lines) and for supernatant progeny virus by the 50% tissue culture infectious dose (TCID_50_) assay (lower lines). BTV2 PBMC = BTV2 VP7 RNA in PBMC, BTV12 PBMC = BTV12 VP7 RNA in PBMC, BTV2 PT = BTV2 TCID_50_ in PBMCs, and BTV12 PT = BTV12 TCID_50_ in PBMCs. Both infection conditions of BTV2 and BTV12 are at a multiplicity of infection (MOI) of 1. * *p* < 0.05 indicates a significant difference between BTV values at the same time points.

The yields of PBMCs were approximately 4.52 ± 1.7 × 10^6^ cells/mL in a 25 mL suspension derived from 50 mL blood (*n* = 8). The standard curve of absolute viral loads in PBMCs was quantified by qRT-PCR using an external BTV VP7 cRNA standard [7]. The detection limit of 10^1^ copies/μL was previously described [7]. The VP7 RNA copy numbers and titers in tissue culture infectious dose (TCID_50_) are presented in Figure 2. 

**Figure 3:**
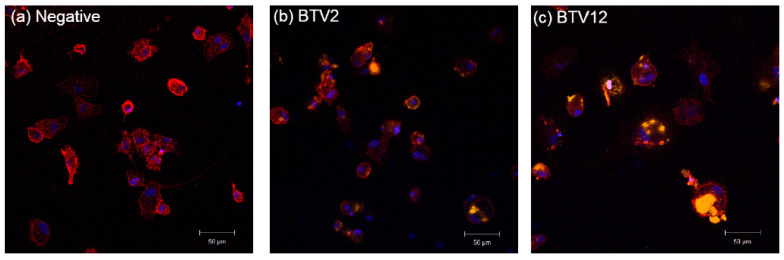
BTV2- and BTV12-infected monocyte-derived macrophages (MDMs) observed under confocal microscopy. (**a**) Negative control, (**b**) infected with BTV2 (MOI 1), (**c**) infected with BTV12 (MOI 0.5). Macrophage surface marker CD172a was labeled with the mouse anti-bovine CD172a antibody, with a secondary antibody of anti-mouse IgG1 conjugated with PerCP/Cyanine5.5 (Red). The BTV signal was labeled with the rabbit anti-BTV-NS3 antibody with a secondary antibody of goat anti-rabbit IgG conjugated with Alexfluor 546 (Orange). The cell nucleus was labeled with 4′,6-diamidino-2-phenylindole (DAPI, Blue). Uninfected MDMs express membranous CD172a with a more irregular cell shape. The BTV-infected MDMs express cytoplasmic NS3 protein with a more rounded shape.

The intracellular loads of BTV2 were 10^3.35^ copies/μL at 0 hpi and peaked at 10^4.04^ copies/μL at 12 hpi. The supernatant progeny virus of BTV2 was 10^1.63^ TCID_50_/mL at 0 hpi and peaked at 12 hpi with 10^2.69^ TCID_50_/mL. The intracellular loads of BTV12 were 10^3.45^ copies/μL at 0 hpi and peaked at 10^4.47^ copies/μL at 12 hpi. The supernatant progeny virus of BTV12 was 10^1.63^ TCID_50_/mL at 0 hpi and peaked at 12 hpi (10^3.22^ TCID_50_/mL). Overall, the viral loads of both BTV2 and BTV12 were similar, with a higher load of BTV12 than BTV2 at 12 hpi, 2.69-fold higher VP7 RNA, and 3.39-fold higher TCID_50_ (Figure 2), consistent with the observation in Figure 3 (Section 2.3). 

### 2.3. Infection Rate of BTV2 and BTV12 in MDMs Detected by IFA

After 7 days of cultivation, the yields of MDMs were approximately 1 ± 0.4 × 10^5^ cells/mL/well in a 12-well plate. Uninfected bovine MDMs expressed membranous CD172a (red) with a more irregular cell shape (Figure 3a). The infected MDMs exhibited both membranous CD172a (red) and an intracytoplasmic viral NS3 signal (orange), with a more rounded cell shape (Figure 3b,c). The infection rate of BTV2, at multiplicity of infection (MOI) 1, was 75% (*n* = 80 cells counted, Figure 3b). When MDMs were infected by BTV12, at MOI 1, almost all cells died and detached. This was consistent with the finding in Figure 2. The infection rate of BTV12 was only countable at MOI 0.5, which was 76.1% (*n* = 46, Figure 3c). 

### 2.4. Expression of mRNA in BTV-Infected PBMCs

**Figure 4:**
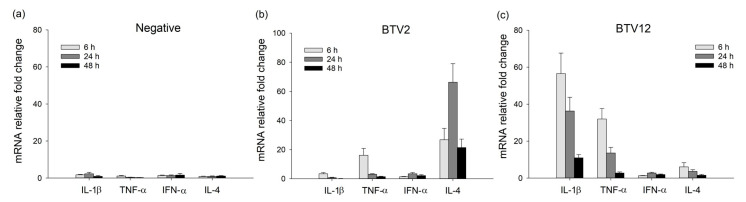
The differential effects of BTVs on the cytokine expression in PBMCs. (**a**) Negative control, (**b**) BTV2 (MOI 1), and (**c**) BTV12 (MOI 1). *p* < 0.05 indicates a significant difference between BTVs and negative control values at the same time points.

BTV2 induced marked IL-4 expression early at 6 hpi, and it was significantly higher than that of BTV12 and negative groups at 6–48 hpi (Figure 4a,b), with a low significant expression of TNF-α only being observed at 6 hpi. Both results were consistent with our previous data [7]. 

In contrast, the expression of IL-1β in the BTV12 group peaked at 6 hpi (56.554-fold, *p* < 0.01), and was significantly higher than the values of other groups, and then gradually declined up to 48 hpi (Figure 4c). Meanwhile, IL-4 increased only slightly, with its maximum at 6 hpi (6.031-fold, Figure 4c). The level of TNF-α induced by BTV12 peaked at 6 hpi (31.947-fold, *p* < 0.05), and was significantly higher than the values of the BTV2 group and negative control, and then gradually declined by 48 hpi (Figure 4c).

Overall, the mRNA expression of IL-1β and TNF-α of the BTV12 group was strongly activated, whereas that of the IL-4 was minimal and significantly lower than that of the BTV2 group. IFN-α of the BTV2 group peaked at 24 hpi (3.465-fold), and that of the BTV12 group also peaked at 24 hpi (2.759-fold) (Figure 4b,c). 

### 2.5. The Cytokine Production Induced by BTV2 and BTV12

**Figure 5:**
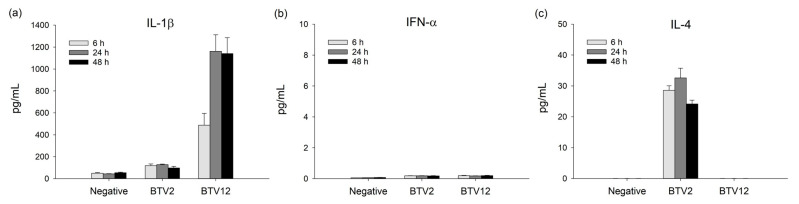
The differential effects of BTVs on the cytokine production in PBMCs. (**a**) Interleukin-1 (IL-1)β, (**b**) interferon-α (IFNα), and (**c**) IL-4. Both infection conditions of BTV2 and BTV12 are MOI 1. *p* < 0.05 indicates a significant difference between BTVs and negative control values at the same time points. These data are consistent with the mRNA expression profile (Figure 4).

The IL-1β in the BTV12 group showed a sustained significantly high production at 24–48 hpi and then peaked at 1162.02 pg/mL (*p* < 0.01) at 24 hpi (Figure 5a). The BTV-12-induced IL-1β production was significantly greater than that in BTV2 and the negative control (Figure 5a), which was consistent with the mRNA expression (Figure 4c). On the other hand, the protein levels of IFN-α were consistently low (the detection limit was 15 pg/mL, Figure 5b), in line with their low mRNA expressions (Figure 4b,c). 

In contrast, the IL-4 production in the BTV12 group remained below the detection limit at all time points (Figure 5c), whereas that of the BTV2 group was significantly and persistently high during 6–48 hpi and peaked at 32.60 pg/mL (*p* < 0.05) at 24 hpi (Figure 5c), consistent with the mRNA expression (Figure 4b).

### 2.6. Prediction of Potential IL-4-Inducing Peptides on the VP2 of Various BTV Strains

IEDB analysis showed that those BTVs (serotypes 2, 8, 11, and 17) with records of an allergy, manifested by IL-4 expression, had significantly higher numbers of potential IL-4-inducing peptides (segments) on the whole length of VP2 than those BTVs (serotypes 12, 1) without allergy records (Table 1 and Table S1), averaging 24.80 ± 2.713 segments (*n* = 20) versus 19.80 ± 2.926 segments (*n* = 15) (*p* < 0.01), respectively. 

**Figure 6:**
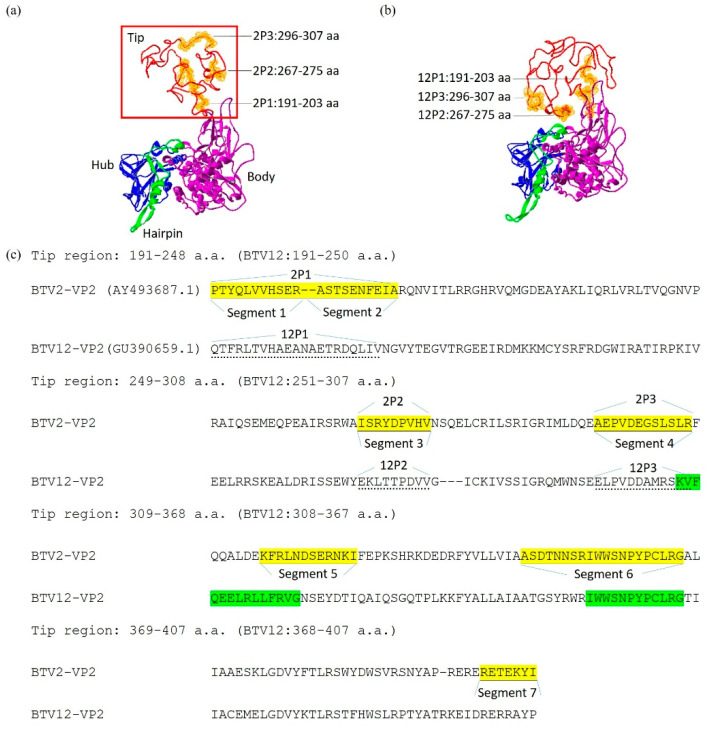
The 3D structure of the VP2 protein of the four BTV serotypes (BTV2, 12, 11, and 1, Table 1 and Table S2) indicates distinct folding in the tip region (boxed), while the remaining structures are strikingly similar. (**a**) BTV2 (AY493687.1), where 2P1 to 2P3 are the 3D locations of synthesized segments of BTV2. (**b**) BTV12 (GU390659.1), where 12P1 to 12P3 are the 3D locations of synthesized segments of BTV12. (**c**) The alignment of the VP2 tip region sequence of the potential IL-4-inducing peptides selected for testing in PBMCs. Solid underlined sequences indicate the seven segments (yellow shaded) of predicted IL-4-inducing peptides in the tip region of VP2 of BTV2, and the two segments (green shaded) presented on that of BTV12. Segments 1–2–3–4 (2P1-2-3) of BTV2 were selected for a separate synthesis to form a cocktail (or a copy), while their corresponding sequences of BTV12 (12P1-2-3, dotted underlined) were synthesized for a comparison of their capability to induce IL-4.

Modeling of the 3D structure of the VP2 of BTV2 and BTV12 showed their folding in the tip region to be distinct from one another (Figure 6a,b), while the rest of the VP2 mainframe structure was similar. This distinct folding in the tip region may be related to their capability to interact with the host receptor, in this case, on PBMCs. There were higher numbers of potential IL-4-inducing peptides, averaging 7.20 ± 0.748 segments (*n* = 20), in this VP2 tip region of allergic BTVs, compared to the 2.60 ± 0.800 segments (*n* = 15) (*p* < 0.01) recorded for BTVs without allergy records (Table 1). That of the non-allergy strain BTV10 was graded intermediate (Table 1 and Table S1).

### 2.7. Predicted IL-4-Inducing Peptide Segments in the VP2 Tip Region of BTV2 and BTV12

The tip region of VP2 of BTV2, having significant IL-4 expression (Figure 4b and Figure 5c), contained seven segments (numbers 1–2–3–4–5–6–7, Figure 6a,c) of the potential IL-4-inducing peptide (yellow shaded, Figure 6c), whereas that of BTV12 contained only two segments (numbers 1–2, shaded green, Figure 6b,c; Table S2). The ratio of the total copy number of synthetic peptides to PBMC was set at 100 copies (=400 segments) per PBMC, which approximated the dose of 10^2^ TCID_50_ at 6 hpi (Figure 2), and each segment was assumed to have an equal IL-4-inducing capability.

To test whether this segment number difference accounted for the different immune responses observed above, cocktails of four segments of potential IL-4-inducing peptides (Figure 6c) were synthesized to stimulate the PBMCs.

### 2.8. Induction of IL-4 Production in PBMC Stimulated with Synthetic Peptide

According to the instructions from the manufacturer, standard curves of IL-4 protein with the cover range of the IL-4 concentration ranging from 8.19 to 800 pg/mL were determined in our previous experiments (the R^2^ value was 0.9921) [7].

**Figure 7:**
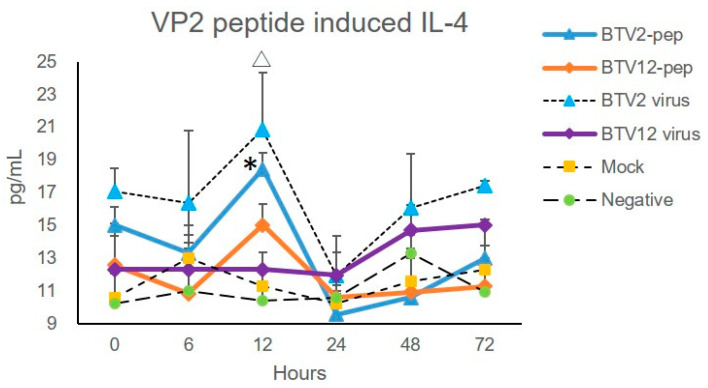
Cocktails of synthetic peptides on VP2 of BTV2 induced significant IL-4 protein production in PBMCs. BTV2-pep: BTV2 peptide cocktail and BTV12-pep: BTV12 peptide cocktail. Both infection conditions of BTV2 and BTV12 are at MOI 1. * and △ *p* < 0.05 indicate a significant difference between BTV2-pep (as well as BTV2 virus) and the other four groups, i.e., BTV12-pep, BTV12 virus, mock, and negative control values, at the same time points.

The IL-4 in the BTV2-pep group displayed a significantly high expression of 18.437 pg/mL at 12 hpi, as did that induced by BTV2 virus, which also peaked at 12 hpi (20.827 pg/mL, Figure 7). Both were significantly higher than those of the other four experimental groups (*p* < 0.05), consistent with the prediction (Figure 6; Table 1) and mRNA expression profile (Figure 4). There were no significant differences between the responses to BTV2-pep and BTV2 virus (*p* = 0.128).

The IL-4 in the BTV12-pep group also peaked at 12 hpi (15.023 pg/mL), and IL-4 in the BTV12 virus group peaked at 72 hpi (15.023 pg/mL, Figure 7). Both were significantly lower than those of the BTV2-pep group.

## 3. Discussion

This study demonstrates the different responses of bovine PBMCs (summarized in Table 2) induced by the two Taiwanese strains BTV2/KM/2003 and BTV12/PT/2003. The replication curve (Figure 2) and infection yield (Figure 3) of both BTV2 and BTV12 were largely parallel in both PBMCs and MDMs, yet the cytokine profiles they induced were significantly different (Figure 4b,c and Figure 5a,c). The infection rate of BTV12 can only be calculated at MOI 0.5, suggesting that BTV12 may have a stronger affinity or virulence to PBMC monocytes and MDMs (Figure 2), in which infected monocytes/MDMs activated the innate immunity (Figure 4c), as is normally expected for sequential immune responses [17,18]. These infected monocytes/MDMs then underwent necrosis/lysis (Figure 3c), resulting in a reduced number of intact cells available for analysis (Figure 3c and data not shown). 

**Figure 8:**
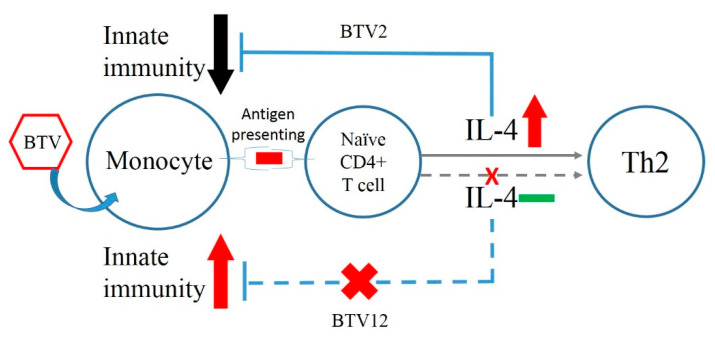
Different immune pathways induced by BTV2 and BTV12 in bovine PBMCs. BTV2 induces significant IL-4 expression and then feeds back to inhibit innate immunity (upper part) [7]. In contrast, BTV12 activates innate immunity strongly, without inducing significant IL-4 expression.

As in regular viral infection, BTV12 activated monocytes normally and strongly, indicated by the TNF-α and IL-1β expressions, but activated the Th2 pathway poorly, indicated by the IL-4 (Figure 4c, Figure 5c and Figure 8). In contrast, BTV2 infection activated innate immunity poorly but activated the Th2 pathway significantly (Figure 4b). On the other hand, BTV2 seemed able to infect monocytes (Figure 3b) while activating the Th2 pathway early on (Figure 4b), which then fed back to inhibit the innate immunity (Figure 4b and Figure 8) [7,19]. These differences observed between BTV2/KM/2003 and BTV12/PT/2003 (Figure 8) were also found in [4], wherein BTV1 stimulated a significant IL-1β expression, but BTV8 did not. It is speculated that the abolition of IL-1β activation might be key for BTV survival in infected animals [20]. 

The type I interferon was minimally expressed at both the mRNA and protein levels in both BTV2- and BTV12-infected PBMCs (Figure 4b,c and Figure 5b) in the present study setting. The result contradicts the finding in [14] but is consistent with others showing that BTV utilizes NS3 [15] and NS4 [16] to inhibit type I interferon expression. The NS4 amino acid sequences of Taiwanese strains [10] were similar to the NS4 consensus sequence of [16], suggesting that the NS4 of BTV Taiwanese strains may have contributed to the inhibition of IFNα mRNA transcription in the present study.

Since the two BTV Taiwanese strains have a striking similarity in all proteins, expect VP2 [10], we speculated that VP2, rather than VP5, of BTV2 may be related to the significant IL-4 induction (Figure 4b, Figure 5c and Figure 8) [7]. However, despite the amino acid similarity of only 25.8% on VP2 between the two Taiwanese BTVs, their 3D structures were strikingly similar in the main frame (body, hairpin, and hub) among the serotypes analyzed, and only their tip regions differed significantly (Figure 6) [9]. This allowed us to focus on analyzing the difference between the tip regions, 191–407 a.a., of the two Taiwanese BTVs (Figure 6c).

The number of predicted IL-4-inducing peptide segments contained in the tip region of VP2 seems to be positively correlated with the capability to induce the bovine allergy reported in the literature (Table 1), as well as with the induction of IL-4 expression in the current experimental setting (Figure 4b,c and Figure 5c) [7]. BTV2, which contains seven segments in the tip region (Figure 6, Table 1 and Table S2), is closer to that in BTV11 (with five segments) with reported bovine IgE induction [6]. In contrast, the BTV12 tip region, which only has two segments (Figure 6), is closer to that in BTV1 (with three segments, Table S2), without an allergy record. 

Dhanda et al. showed that 47 MHC alleles induce IL-4 [13]. The charged residues preferentially occupy positions 2, 5, 9, 10, and 15 in the IL-4-inducing peptide sequence, while the aliphatic and aromatic residues are mainly located in the 1st, 2nd, 5th, and 6th positions. BTV2, BTV8 [2], BTV11 [5], and BTV17 [6] have 4–5 more segments than BTV1, 10, and 12 (7.2–2.6 as shown in Table 1). These amino acid differences may account for the differences in the cytokine expressions induced by BTV2 and BTV12.

In general, the IL-4 expressions of PBMCs induced by synthetic peptides of VP2 are similar to those stimulated by virus particles (Figure 7). IL-4 can be induced in both synthetic peptides and virus infection groups. Because the peptides have no replication ability and are only given once, the results of the peptide stimulation test suggested the following three possibilities: 1. IL-4 stimulation may not need a full replication of the virus; 2. synthetic peptides may serve as a good model for inducing hypersensitivity quickly in PBMCs, without requiring large animals; and 3. most events were evident during the early infection. It was easier to distinguish the Th2-inducing capacity of BTV2 and BTV12 by the total IL-4 expression at both mRNA and protein levels, as well as in the peptide induction model. Together, the IL-4-inducing peptides and bovine PBMCs seem to be a good model for pursuing BTV-induced allergic reactions, although the protocol needs further optimization. 

## 4. Materials and Methods 

### 4.1. Animals, and Preparation of Bovine PBMC and Monocyte-Derived Macrophage (MDMs)

Eight 5-year-old female Holstein cows (C1 to C8) in the dry period of the lactation cycle raised in the National Taiwan University (NTU) experimental herd (non-closed environment, the experiment permission number in NTU is IACUC#A02041) were used for blood collection. The whole blood was used for PBMC isolation and BTV infection. All cows were healthy, without clinical signs or medication. All cow sera were free of anti-BTV antibody production, tested by commercial cELISA (VMRD, Pullman, WA, USA) during the 3-year experiment period. Heparinized blood was collected for PBMC isolation and quantitated as previously described [7]. Differentiation of the MDMs in vitro was carried out as previously described [7]. The morphology of MDMs was confirmed via bright field inverted microscopy. The identity of MDMs was demonstrated by macrophage surface marker CD172a (mouse anti-bovine CD172a antibody, Bio-Rad, Hercules, CA, USA) [7] and observed via confocal microscopy. 

Fetal bovine serum (FBS), Roswell Park Memorial Institute (RPMI) 1640 medium, Dulbecco’s modified Eagle medium (DMEM), Hank’s balanced salt solution (HBSS), and phosphate-buffered saline (PBS) were purchased from Thermo (Waltham, MA, USA). Histopaque 1.083 Ficoll medium was purchased from Sigma-Aldrich™ (St Louis, MO, USA).

### 4.2. Virus Infection and Quantitation of BTV Viral Loads in PBMCs

The BTV2/KM/2003 and BTV12/PT/2003 Taiwanese isolates [21] were propagated in seven passages in baby hamster kidney (BHK-21) cells and adjusted to the titer of 10^6^ TCID_50_/mL.

The MDMs and PBMCs were infected with either BTV2 or BTV12 at a multiplicity of infection (MOI) of 1 in triplicate or served as a negative control. The PBMCs were harvested at various hpi and quantitated by qRT-PCR for the mRNA expression levels [7] with primer sequences designed for housekeeping gene ribosomal protein S9 (*RPS9*) [22] and BTV VP7 [7]. The BTV viral loads in PBMCs at various time points were quantified by qRT-PCR for intracellular VP7 RNA expression as in previous research [7]. The BTV-VP7 cRNA standard preparation protocols were also as described in previous research [23]. 

### 4.3. TCID_50_ Assay to Determine the Titer of Progeny Virus in the PBMC Supernatant

The culture supernatants of BTV2- and BTV12-infected PBMCs, harvested at various similar time points, were titrated by the TCID_50_ assay to confirm the progeny virus of BTVs [24,25]. The BHK-21 cells pre-seeded in 96-well plates, at 8 × 10^4^ cells/well, were inoculated with serially diluted samples, from 10^−1^ to 10^−7^, with each being placed into 6 wells (hexaplicate). After 1 h incubation, cells were washed with PBS, fed with DMEM, and cultured during 72 hpi, at which point, cells were washed with PBS, fixed with 95% ethanol, and stained with 0.2% crystal violet in 2% ethanol. To confirm the titer of progeny virus, the cytopathic effects during 1 h incubation (CPE, i.e., necrosis, swelling, and rounding) were scored under 20× magnification using a dissecting microscope. The difference in logarithms of the TCID_50_ assay was calculated by the following formula: [(mortality at dilution next above 50%) − 50%]/[(mortality next above 50%) − (mortality next below 50%)] [24,25].

### 4.4. Pair-Wise Comparison of the Cytokine mRNA Expression by qRT-PCR

The PBMCs were harvested at various hpi and quantitated by qRT-PCR for the mRNA expression levels [7] with primer sequences designed for TNF-α, IL-1β [22], IFN-α [26], and IL-4 [27]. SYBR Green qRT-PCR is a widely used method for investigating cytokine mRNA expression in ruminants [27]. The extraction of total RNA and data analysis were carried out as in our previous research [7]. The Ct values of *RPS9* in qRT-PCR were stable from 16.96 to 19.71. All cytokines of the uninfected negative control group were under the detected limit. Every cytokine Ct was double normalized by housekeeping gene ribosomal protein S9 (*RPS9*) and its 0 hpi to monitor variations in cell health and the validity of the experimental procedures. The “relative” fold change in the cytokine mRNA level was calculated by the 2^−ΔΔCt^ method [28]. The first normalization was employed to calculate ΔCt: each target cytokine mRNA Ct − RPS9 Ct = ΔCt. The second normalization was applied to calculate ΔΔCt: The ΔCt of 6, 24, 48 hpi − the ΔCt of 0 hpi. The relative fold change value was 2^−ΔΔCt^.

### 4.5. IFA of BTV-Infected MDMs by Confocal Microscopy

All MDMs were cultivated on round coverslips within 12-well plates in the 5% CO_2_ incubator at 37 ℃. The MDMs were infected with either BTV2 (MOI = 1) or BTV12 (MOI = 0.5) for 2 h. After 2 h incubation, the culture supernatants were replaced with fresh medium and harvested 12 hpi. To decrease the non-specific binding of antibodies with Fc receptors on MDMs, azide-free Fc receptor blocker (20 μL/10^5^ cells, Innovex Biosciences Inc., Richmond, CA, USA) was used for blocking for 30 min. After blocking, all cells were fixed with 4% paraformaldehyde (PFA, Merck, Darmstadt, Germany) in PBS for 3 min. 

The IFA staining protocol was as described in the previous study [7]. Macrophage surface marker CD172a was labeled with the mouse anti-bovine CD172a antibody, with a secondary antibody of anti-mouse IgG1 conjugated with PerCP/Cyanine5.5 (Rat IgG, BioLegend, San Diego, CA, USA). BTV signals were labeled by rabbit anti-BTV-NS3 antibody (GenScript, Piscataway, NJ, USA), with a secondary antibody of goat anti-rabbit IgG conjugated with Alexfluor 546 (Thermo, Waltham, MA, USA). After staining, DAPI-Fluoromount-G Mounting Medium (Invitrogen, Carlsbad, CA, USA) was used to mount the slide. Cells were microphotographed with a Zeiss LSM 780 confocal microscope by using ZEN 2009 Light Edition software (Carl Zeiss, Oberkochen, Germany).

### 4.6. Enzyme-Linked Immunosorbent Assay (ELISA) for IL-1β, IFN-α, and IL-4

The supernatants of PBMCs infected with BTVs were harvested at each time point to monitor cytokine production. The commercial bovine IL-1β ELISA kit was purchased from Thermo Inc. (Waltham, MA, USA) and bovine IL-4 ELISA kit was purchased from Sigma-Aldrich™ (St Louis, MO, USA). Their standard curves were established as previously described [7]. The commercial IFN-α ELISA kit was purchased from Sunlong Inc. (Hangzhou, Zhejiang, PRC) and its standard curve was established as shown in Figure S1.

### 4.7. The Prediction of Potential IL-4-Inducing Peptides and Three-Dimensional (3D) Protein Structure Modeling on VP2 of BTVs

Potential IL-4-inducing peptides in the VP2 tip region were predicted by the IL4pred online platform for IL-4 motif scanning (https://webs.iiitd.edu.in/raghava/il4pred/). The BTV-VP2 protein sequences in FASTA format [29] were entered in the “Scanning of IL-4 Motifs” field, and the Koolman–Röhm class of motifs [30] was selected for searching and visualizing the motifs tabulated (Table 1; Tables S1 and S2). After the database server replied, repeat sequence items were removed, and both the total number of IL-4-inducing peptides in the tip region (191–407 a.a.) and the full length of VP2 were calculated manually.

Three-dimensional protein structure models of VP2 was generated by the SWISS-MODEL homology-modeling pipeline (https://swissmodel.expasy.org/). The IL-4 motif sequence predicted by the IL4pred was located in the 3D model. The IL-4 motif location in the outermost area of the tip region was selected for ex vivo testing on bovine PBMCs.

### 4.8. Synthesis of BTV Potential IL-4 Inducing Peptides

For BTV2-VP2: Segment 1–2 (2P1) is PTYQLVVHSERASTSENFEIA at amino acid (a.a.) 191–211 in the VP2 tip region; segment 3 (2P2) is ISRYDPVHV at a.a. 267–275 in the VP2 tip region; and segment 4 (2P3) is AEPVDEGSLSLR at a.a. 296–307 in the VP2 tip region. For BTV12-VP2: 12P1 is QTFRLTVHAEANAETRDQLIV at a.a. 191–213 in the VP2 tip region; 12P2 is EKLTTPDVV at a.a. 269–277 in the VP2 tip region; and 12P3 is ELPVDDAMRSKV at a.a. 295–306 in the VP2 tip region. The peptides were synthesized by GenScript (Piscataway, NJ, USA) and Academia Sinica (Taipei, Taiwan, ROC). The purity of all peptides was 95%. The synthetic peptides were dissolved in PBS (1 mg/mL) and stored at −25 °C.

### 4.9. PBMC Stimulation Assay for Synthetic IL-4-Inducing Peptides

The PBMC stimulation assay was modified from the reference [31]. BTV-free bovine PBMCs were isolated from another three 5-year-old Holstein cows (designated NC 4–6). The new PBMCs were divided into six groups: BTV2-pep (induced with peptide cocktail 2P1–3, 0.5 ng/well/1 × 10^6^ PBMC); BTV12-pep (peptide cocktail 12P1–3, 0.5 ng/well/1 × 10^6^ PBMC); BTV2 virus (MOI 1); BTV12 virus (MOI 1); BHK21 lysates (mock control); and uninfected negative control. The peptide cocktail contained a full set of segments 1–2–3–4 for BTV2 and their corresponding sequences at the same locations for BTV12. 

All PBMC groups were harvested at 0, 6, 12, 24, 48, and 72 hpi. The supernatants of each hpi were tested for IL-4 protein levels by ELISA. All data were represented by the averages from nine wells (three cows each with triplicate wells). 

### 4.10. Statistical Analysis

The data in Figure 2, Figure 4, Figure 5 and Figure 7 were analyzed by two-way ANOVA in SPSS version 20.0 (IBM^®^, Armonk, NY, USA) as previously described [7]. The data in Table 1 were analyzed by one-way ANOVA in SPSS version 20.0 (IBM^®^, Armonk, NY, USA).

## 5. Conclusions

Although both growth curves of BTV2 and BTV12 are very similar in infected PBMCs, the cytokine profiles they induce differ significantly. BTV2 preferentially activates the Th2 pathway, represented by IL-4 expression, but it poorly activates the innate immunity. In contrast, BTV12 preferentially activates the innate immunity, represented by TNF-α and IL-1β, with only minimal IL-4 expression. The capability to induce IL-4 production is attributed to the tip region on the VP2 protein, and the number of predicted IL-4-inducing peptide segments contained therein is positively correlated with the allergic reaction reported in cattle or the more significant IL-4 expression in ex vivo settings.

## Figures and Tables

**Table 1:** Numbers of Potential IL-4-Inducing Peptide Segments in the Tip Region of VP2 of Allergy-Reported BTVs and Non-Allergy Reported BTVs.

	BTV Serotype	Allergy-Reported	Non-Allergy Reported		
Number		2, 8, 11, 17 (*n* = 20)	12, 1 (*n* = 15)	10 (*n* = 7)	*p* Value (One-Way ANOVA)
Tip region ^a^		7.20 ± 0.748	2.60 ± 0.800	4.286 ± 0.452	<0.01
Total VP2 ^b^		24.80 ± 2.713	19.80 ± 2.926	21.714 ± 1.666	<0.01
Tip/Total ^c^		0.290 ± 0.0469	0.131 ± 0.0462	0.197 ± 0.0104	<0.01

Note: ^a^ Number of potential IL-4-inducing peptide segments in the tip region of VP2. ^b^ Number of potential IL-4-inducing peptides on the whole length of VP2. ^c^ Ratio of number in the tip region divided by number on the whole length of VP2. The raw data are provided in Table S1.

**Table 2:** Differential Responses in Bovine PBMCs Induced by BTV2 and BTV12.

Parameters	BTV2	BTV12
Replication (VP7) in bovine PBMC (Figure 2)	Parallel	Parallel, slightly higher
Replication (TCID_50_) in bovine PBMC (Figure 2)	Parallel	Parallel, more virulent
Infection rates in MDMs (Figure 3)	75% at MOI of 1 at 12 hpi	Cells died and detached at MOI of 1 by 12 hpi; 76.1% at MOI of 0.5. Stronger affinity or virulence to MDM/PBMC
Innate immunity (TNF-α and IL-1β) (Figure 4, Figure 5 and Figure 8)	Minimal to mild	Activated strong and sustained, 2–4-fold higher (TNF-α) at 6–12 hpi 17–27-fold higher (IL-1β) at 6–12 hpi Likely part of a cytokine storm, which is cytocidal (combined Figure 3 with Figure 4c)
Acquired immunity (Th2 and IL-4) (Figure 4, Figure 5 and Figure 8)	Activated strong and sustained 6–11-fold higher	Minimal
Number of potential IL-4-inducing segments in the tip region of VP2 (Figure 6)	7	2
Association with allergy (Table 1, Supplement Tables S1 and S2)	Yes	Low
IL-4-inducing capacity of synthetic peptides (Figure 7)	Yes	Low

## Supplementary Materials

The following are available online at https://www.mdpi.com/2076-0817/10/1/3/s1; Figure S1: Bovine IFN ELISA protein standard curve; Table S1: Number of predicted IL-4-inducing peptide segments in the VP2 tip region and full length among BTV serotypes; Table S2: List of predicted IL-4-inducing peptide sequences in the BTV tip region (191–407 a.a.) among BTV serotypes.
