# Supplementary material for: The Tip Region on VP2 Protein of Bluetongue Virus Contains Potential IL-4-Inducing Amino Acid Peptide Segments
Source: Pathogens. 2020 Dec 22;10(1):3. doi: 10.3390/pathogens10010003 (PMC7822166; doi:10.3390/pathogens10010003)
Supplement: Supplementary file 1 [file pathogens-10-00003-s001.zip › Supplement Table S1.docx]

Table S1 Number of predicted IL-4-inducing peptide segments inthe VP2 tip region and full length among BTV serotypes

| IgE report (**+/−**) | Serotype | Accession number | IL-4 inducing peptides (number) | | Common starting site(a.a.) | References |
| --- | --- | --- | --- | --- | --- | --- |
|  |  |  | Tip region | Total region |  |  |
| **+** | 2^a^ | AY493687.1 (Taiwan)  AB686224.1 (Japan)  AJ585152.1 (India)  AY855265.1 (US)  AJ585123.1 (South Africa)  DQ191261.1 (Italy)  KP821024.1 (France)  JQ240322.1 (Australia) | 7  8  8  7  7  7  7  8 | 24  26  28  27  27  27  27  28 | 191, 201, 296,  315, 344, 397 | Lee et al., 2011  Shirafuji et al., 2012  Maan et al., 2004  Mecham et al., 2005  Maan et al., 2004  Potgieter et al., 2005  Nomikou et al., 2015  Boyle et al., 2012 |
|  | 8^b^ | JX272540.1 (South Africa)  KP821072.1 (Israel)  KP821073.1 (Greece)  KU569991.1 (France) | 7  7  7  7 | 25  23  24  23 | 256, 333, 346, 398 | Koekemoer et al.,‎ 2012 (Unpublished)  Nomikou et al., 2015  Nomikou et al., 2015  Breard et al., 2016 |
|  | 11^c^ | JQ972852.1 (Germany)  KM580420.1 (US)  JN003580.1 (France)  JX272510.1 (South Africa) | 8  6  5  8 | 25  29  25  26 | 256, 314, 343, 386 | Vandenbusscheet al.,‎ 2015  Gaudreault et al.,‎ 2015  Viarouge et al.,‎ 2011 (Direct submission)  Koekemoer et al.,‎ 2012 (Unpublished) |
|  | 17^d^ | KX599360.1 (Brazil)  AY855269.1 (US)  JX272450.1 (South Africa)  AJ585138.1 (South Africa) | 8  8  7  7 | 22  21  20  19 | 196, 251, 314, 329, 343 | Matos et al.,‎ 2014 (Unpublished)  Mecham et al.,‎ 2005  Koekemoer et al.,‎ 2012 (Unpublished)  Maan et al., 2004 |
| **−** | 12 | GU390659.1 (Taiwan)  KX164080.1 (USA)  AJ585185.1 (Kenya)  AB686216.1 (Japan)  KC662613.1 (India)  JX272500.1 (South Africa)  KP821100.1 (South Africa)  AJ585133.1 (South Africa) | 2  2  2  2  2  2  2  2 | 22  19  22  22  19  21  21  21 | 308,348 | Lee et al., 2011  Johnson et al., 2016(Direct submission)  Maan et al., 2003 (Unpublished)  Shirafuji et al., 2012  Rao,P.P. et al., 2013  Koekemoer et al.,‎ 2012 (Unpublished)  Nomikou et al., 2015  Maan et al., 2004 |
|  | 1 | 3J9D  FJ437557.1 (France)  KM099539.1 (Australia)  KP821018.1 (Spain)  KP821022.1 (Sudan)  KX164020.1 (USA)  KF664124.1 (India) | 3  3  3  3  3  3  5 | 15  15  24  18  15  19  24 | 311, 345, 386 | Zhang et al., 2016  Cetre-Sossah et al., 2011  Boyle et al., 2014  Nomikou et al., 2015  Nomikou et al., 2015  Johnson et al., 2016 (Direct submission)  Ranjan et al., 2013 |
|  | 10 | KX247939.2 (Canada)  JX272520.1 (South Africa)  AJ585131.1 (South Africa)  KT317696.1 (South Africa)  JQ740772.1 (India)  JN704634.1 (India)  KP339245.1 (India) | 5  5  4  4  4  4  4 | 23  25  21  22  21  20  20 | 269,314,343,386 | Nishi et al., 2016 (Direct Submission)  Koekemoer et al.,‎ 2012 (Unpublished)  Maan et al., 2004  Van den Berghet al., 2015(Unpublished)  Maanet al., 2012  Anshulet al., 2011 (Unpublished)  Reddy et al., 2014 (Unpublished) |

Note: Unpublished and Direct submissionsequences are not under the list of references.
