# Supplementary material for: The Tip Region on VP2 Protein of Bluetongue Virus Contains Potential IL-4-Inducing Amino Acid Peptide Segments
Source: Pathogens. 2020 Dec 22;10(1):3. doi: 10.3390/pathogens10010003 (PMC7822166; doi:10.3390/pathogens10010003)
Supplement: Supplementary file 1 [file pathogens-10-00003-s001.zip › Supplement Table S2.docx]

Table S2 List of predicted IL-4-inducing peptide sequences in the BTV tip region (191-407 a.a.) among BTV serotypes

| BTV Serotype | Sequence | Site (a.a.) | Motif (Koolman-Rohmclassification) |
| --- | --- | --- | --- |
| 2 (IgE+)^a^ | PTYQLVVHSERAS  RASTSENFEIA  ISRYDPVHV  AEPVDEGSLSLR  KFRLNDSERNKI  ASDTNNSRIWWSNPYPCLRG  RETEKYIFSRINL | 191-203  201-211  267-275  296-307  315-326  347-366  401-413 | E gap I gap P gap aliphatic gap neutral  Basic gap neutral acidic gap F gap aliphatic  Aliphatic gap neutral gap aromatic gap P gap basic aliphatic  A gap acidic gap neutral gap L gap aliphatic basic  K aromatic gap acidic gap basic gap aliphatic  Aliphatic gap neutral gap aromatic gap P gap basic aliphatic  Aliphatic gap neutral gap aromatic gap P gap basic aliphatic |
| 12 | KVFQEELRLLFRVG  IWWSNPYPCLRG | 310-323  360-371 | Basic gap neutral acidic gap F gap aliphatic  Aliphatic gap neutral gap aromatic gap P gap basic aliphatic |
| 11 (IgE+)^b^  (KM580420.1) | ITNSERNNTEETYAPGVHNRI  RTEWINAQFDSTKI  KTDSEHINIFNVGAPA  VWRTNPYPCLRG  KWSLRPDYGRTEVPL | 195-215  261-274  317-332  354-365  386-401 | Aliphatic gap neutral gap aromatic gap P gap basic aliphatic  Basic gap neutral acidic gap F gap aliphatic  E gap I gap P gap aliphatic gap neutral  Aliphatic gap neutral gap aromatic gap P gap basic aliphatic  K aromatic gap acidic gap basic gap aliphatic |
| 1  (3J9D) | KFRQHDPERLKI  VWWSNPYPCLRG  YDWSVRPTYTPYEK | 315-326  355-366  386-399 | K aromatic gap acidic gap basic gap aliphatic  Aliphatic gap neutral gap aromatic gap P gap basic aliphatic  Y D gap neutral gap K |

^a^IgE production by BTV2 challenged bovine PBMC, Yang et al., 2020.

^b^IgE production by BTV11 challenged bovine PBMCin experimental animal infection, Odeon et al., 1999.
