# Supplementary figures and images for: The Tip Region on VP2 Protein of Bluetongue Virus Contains Potential IL-4-Inducing Amino Acid Peptide Segments
Source: Pathogens. 2020 Dec 22;10(1):3. doi: 10.3390/pathogens10010003 (PMC7822166; doi:10.3390/pathogens10010003)

Fig. S1 Bovine IFNα ELISA protein standard curve


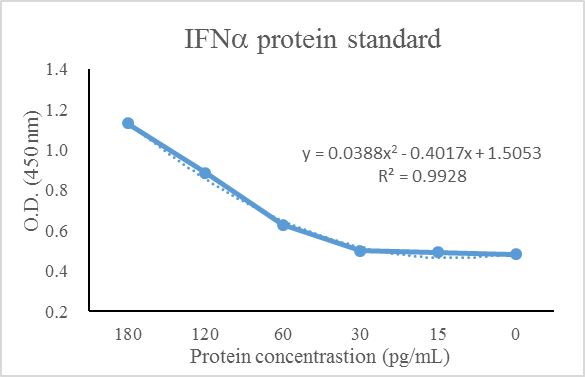

Supplement: Supplementary file 1 [file pathogens-10-00003-s001.zip › Supplement Fig. S1.docx]
